# Supplementary material for: Advantages of Induced Circular Dichroism Spectroscopy for Qualitative and Quantitative Analysis of Solution-Phase Cyclodextrin Host–Guest Complexes
Source: Int J Mol Sci. 2023 Dec 28;25(1):412. doi: 10.3390/ijms25010412 (PMC10779089; doi:10.3390/ijms25010412)
Supplement: Supplementary file 1 [file ijms-25-00412-s001.zip › IJMS_2776029_Supplementary Material.pdf]

Supplementary Material

Advantages of Induced Circular Dichroism spectroscopy for qualitative and quantitative analysis of solution-phase cyclodextrin host-guest complexes.

Márta Kraszni[1], Balázs Balogh[2], István Mándity[2] and Péter Horváth[1]*

[1] Semmelweis University, Department of Pharmaceutical Chemistry, Hőgyes Endre utca 9, 1092 Budapest, Hungary

[2] Semmelweis University, Department of Organic Chemistry, Hőgyes Endre utca 7, 1092 Budapest, Hungary

*to whom correspondence should be addressed:

Dr. Péter Horváth

Department of Pharmaceutical Chemistry, Semmelweis University

H-1092 Budapest, Hőgyes E. u. 9, Hungary

Phone/Fax: +3612170891

E-mail: horvath.peter@semmelweis.hu

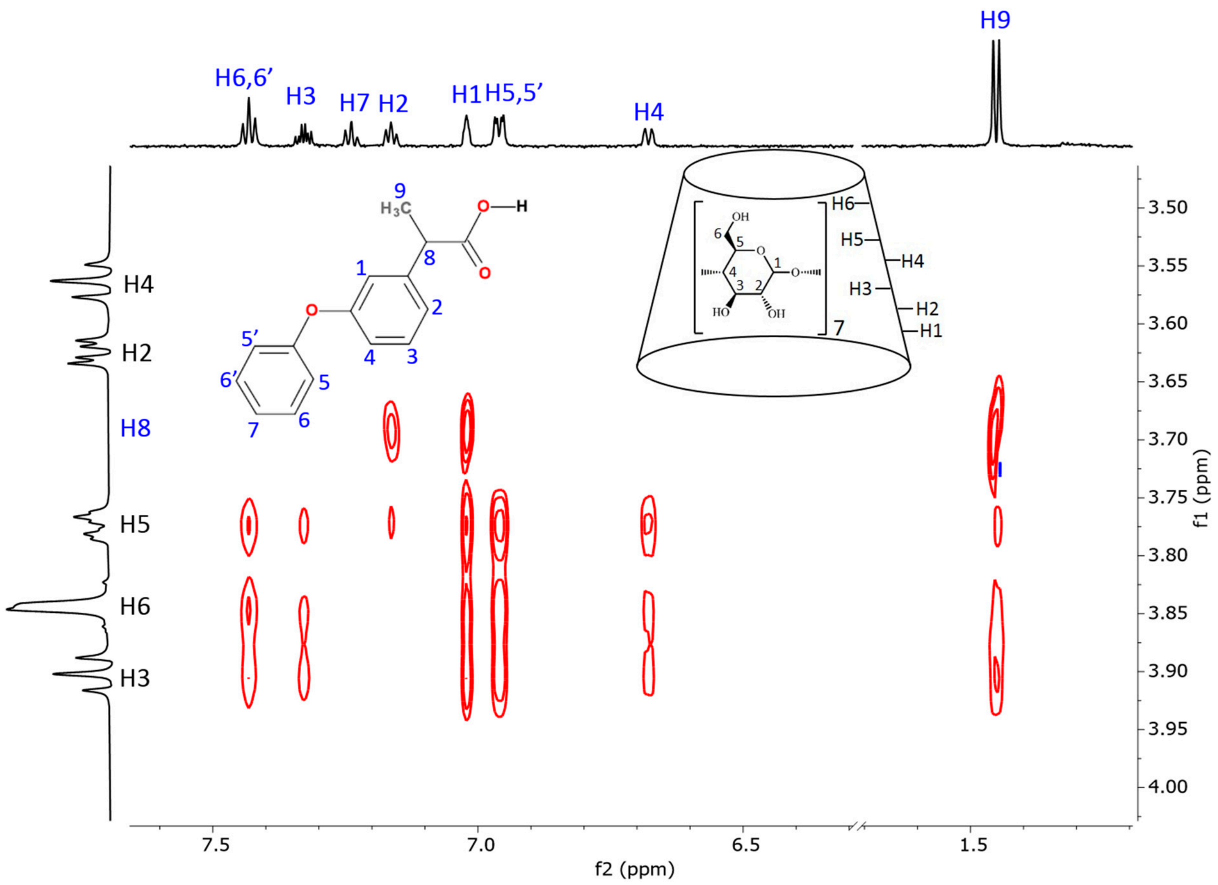

Figure S1 The ROESY NMR spectrum of fenoprofen-BCyD 1:5 concentration ratio in 90% H_2O/10% methanol-d_4. Assignation of the signals in blue is for fenoprofen, in black is for BCyD.
